# Comment on ‘Impact of NICE guidance on tamoxifen prescribing in England 2011-2017: an interrupted time series analysis’

**DOI:** 10.1038/s41416-018-0205-8

**Published:** 2018-08-21

**Authors:** Keith Hopcroft

**Affiliations:** Laindon Health Centre, Basildon, SS15 5TR Essex UK

While I agree that the uptake of NICE guidance by GPs may be slow—and there may be all sorts of reasons for this—I think your recently published paper^1^ highlighting the impact (or lack of) of NICE guidance on tamoxifen prescribing is flawed, and misrepresents GPs.

First, while acknowledging that chemoprevention often takes place in secondary care, you also state that, ‘GPs are also expected to initiate appropriate prescribing’. I would contest this. Your reference in support of this statement states, “Recommendation 6: NHS England should work through CCGs to ensure that GPs are appropriately prescribing chemopreventive agents to reduce the risk of invasive breast cancer where their use is established through NICE guidelines”^2^.

This does not state, and nor do I see it as implying, that GPs should initiate this treatment (I know of no GPs who would do this, whether or not familiar with the guidance). Instead, it suggests that CCGs should (presumably through funding/prescribing monitoring etc) ensure that GPs are following the NICE guidance, which states that ‘Healthcare professionals within secondary care or specialist genetic clinics should discuss the absolute benefits and risks of options for chemoprevention with women at high or moderate risk of breast cancer’^3^ and then offer chemoprevention as appropriate—with the GP taking up the ongoing prescribing (not initiating it).

So to suggest that uptake of NICE guidance by GPs in this area is poor because of a lack of evidence of them initiating the treatment seems to me to be based on a false premise.

Second, you could perhaps suggest that the apparent lack of uptake of chemoprevention might reflect GPs not seeking out those patients with a family history (ie at potentially higher risk) and referring on to secondary care as appropriate. Again, this would be unfounded, given NICE’s unambiguous statement, ‘Healthcare professionals should respond to a person who presents with concerns but should not, in most instances, actively seek to identify people with a family history of breast cancer.’

GPs are used to being blamed for all sorts of issues, sometimes correctly–but this particular paper^1^ critical of GP action (or inaction) seems to me gratuitous and unfounded.
